# Frontotemporal lobar degeneration and social behaviour: Dissociation between the knowledge of its consequences and its conceptual meaning

**DOI:** 10.1016/j.cortex.2017.05.009

**Published:** 2017-08

**Authors:** Roland Zahn, Sophie Green, Helen Beaumont, Alistair Burns, Jorge Moll, Diana Caine, Alexander Gerhard, Paul Hoffman, Benjamin Shaw, Jordan Grafman, Matthew A. Lambon Ralph

**Affiliations:** a Institute of Psychiatry, Psychology & Neuroscience, Department of Psychological Medicine, King's College London, London, SE5 8AZ, UK; b Neuroscience and Aphasia Research Unit, Division of Neuroscience and Experimental Psychology, The University of Manchester, Manchester, UK; c Department of Neurology, Washington University School of Medicine, St. Louis, MO, USA; d Division of Neuroscience and Experimental Psychology, The University of Manchester, Manchester, UK; e Cognitive and Behavioral Neuroscience Unit, D'Or Institute for Research and Education (IDOR), Rio de Janeiro, RJ, Brazil; f National Hospital for Neurology & Neurosurgery, Queen Square, London, UK; g Department of Nuclear Medicine and Geriatric Medicine, University Hospital Essen, Germany; h Rehabilitation Institute of Chicago, Chicago, IL, USA; i Department of Physical Medicine and Rehabilitation, Northwestern University, Chicago, IL, USA

**Keywords:** Brodmann Area 10, Social behaviour, Impulsivity, Disinhibition, Frontal lobe

## Abstract

Inappropriate social behaviour is an early symptom of frontotemporal lobar degeneration (FTLD) in both behavioural variant frontotemporal dementia (bvFTD) and semantic dementia (SD) subtypes. Knowledge of social behaviour is essential for appropriate social conduct. The superior anterior temporal lobe (ATL) has been identified as one key neural component for the *conceptual knowledge* of social behaviour, but it is unknown whether this is dissociable from knowledge of the *consequences* of social behaviour. Here, we used a newly-developed test of knowledge about long-term and short-term consequences of social behaviour to investigate its impairment in patients with FTLD relative to a previously-developed test of social conceptual knowledge. We included 19 healthy elderly control participants and 19 consecutive patients with features of bvFTD or SD and defined dissociations as performance differences between tasks for each patient (Bonferroni-corrected *p* < .05). Knowledge of long-term consequences was selectively impaired relative to short-term consequences in five patients and the reverse dissociation occurred in one patient. Six patients showed a selective impairment of social concepts relative to long-term consequences with the reverse dissociation occurring in one patient. These results corroborate the hypothesis that knowledge of long-term consequences of social behaviour is dissociable from knowledge of short-term consequences, as well as of social conceptual knowledge. Confirming our hypothesis, we found that patients with more marked grey matter (GM) volume loss in frontopolar relative to right superior ATL regions of interest exhibited poorer knowledge of the long-term consequences of social behaviour relative to the knowledge of its conceptual meaning and vice versa (*n* = 15). These findings support the hypothesis that frontopolar and ATL regions represent distinct aspects of social knowledge. This suggests that rather than being unable to suppress urges to behave inappropriately, FTLD patients often lose the knowledge of what appropriate social behaviour is and can therefore not be expected to behave accordingly.

## 1 Introduction

Social knowledge has been defined as knowledge of one's own and other people's minds (Adolphs, 2009). Because “mind” or “mental states” are hard to break down neuropsychologically, we prefer defining social knowledge as denoting non-episodic (i.e., semantic) knowledge of social sensory properties and social behaviour [i.e., functions (Zahn, de Oliveira-Souza, & Moll, 2015)]. Socially appropriate behaviour requires knowledge of adequate social actions within a given short-term sequential context [e.g., ”to appropriately touch a romantic partner after a romantic date” but not “the waiter/waitress after dinner in a restaurant” (Wood & Grafman, 2003)]. Socially appropriate behaviour also requires anticipating possible long-term consequences (e.g., ”being unemployed with a criminal record for sexual harassment” as a consequence of e.g., “inappropriate touching”), but also knowledge of the abstract conceptual quality of a given social action within a given context [e.g., enabling us to flexibly interpret “not being greeted by a colleague who is passing by in a corridor at work” as a sign of “disrespect”, “impoliteness”, “shyness” or “absent-mindedness” (Zahn, Moll, Iyengar et al., 2009)].

Using fMRI and transcranial magnetic stimulation (TMS) methods, we have demonstrated that this abstract conceptual social knowledge, which is independent of the context of actions (Zahn, Moll, Paiva et al., 2009) and emotions (Zahn et al., 2007, Zahn et al., 2009b), is represented in the superior anterior temporal lobe [ATL: (Pobric, Lambon Ralph, & Zahn, 2016)]. FMRI studies have shown that the ATLs, especially within their superior sectors (Skipper et al., 2011, Zahn et al., 2007), are selectively more activated when considering social concepts than they are for non-social concepts (Ross and Olson, 2010, Simmons et al., 2009, Skipper et al., 2011, Zahn et al., 2007), whereas the ventral ATL is equally engaged for social and non-social concepts (Binney, Hoffman, & Lambon Ralph, 2016). Furthermore, right superior ATL activation increased with the richness of detail with which social concepts describe social behaviour (Zahn et al., 2007). This is in keeping with the role of the ATLs in representing coherent conceptual knowledge (Lambon Ralph and Patterson, 2008, Patterson et al., 2007). Frontotemporal lobar degeneration (FTLD) patients with hypometabolism of the right ATL showed selective impairments on social relative to non-social concepts (Zahn, Moll, Iyengar et al., 2009). Furthermore, two rare cases with selective right and left ATL atrophy respectively showed selective impairments on social versus non-social concepts in the right ATL and an impairment of both types of concepts in the left ATL case (Pobric et al., 2016). These results were in keeping with repetitive TMS of the right and left superior ATL, showing a selective slowing of social relative to non-social concepts in the right superior ATL, as well as a slowing of social and non-social conceptual task responses relative to a non-semantic control condition in the left superior ATL (Pobric et al., 2016). These findings were in keeping with a larger body of evidence on graded hemispheric and regional specialisation within the ATLs for different conceptual content (Rice et al., 2015a, Rice et al., 2015b).

Less is known about the representation of sequential social knowledge. Non-social sequential knowledge was associated with lateral frontal neurodegeneration by showing impairments in the hierarchical organisation of non-social scripts as measured by event ordering tasks (Farag et al., 2010). The structured event complex theory (Wood & Grafman, 2003) posited that the prefrontal cortex is a long-term memory store for event/action sequences (also referred to as “scripts”) with the frontopolar cortex (BA10) representing the most sequentially complex information (Moll, Zahn, de Oliveira-Souza, Krueger, & Grafman, 2005). The specific association of the medial frontopolar cortex with complex sequential representations has been corroborated in fMRI studies in healthy control participants by directly modelling the number of events (Krueger et al., 2009) entailed in daily life activities (e.g., going shopping) and their frequency of occurrence (Krueger, Moll, Zahn, Heinecke, & Grafman, 2007), as well as participants' sequential rule knowledge (Wood, Knutson, & Grafman, 2005). Patients with frontal lesions or neurodegeneration which included the frontopolar cortex were shown to perform poorly on tasks that require sequencing daily life activities [i.e., ordering component events into a sequential order (Krueger et al., 2007b, Sirigu et al., 1996)] and structuring events for real-world planning (Goel, Grafman, Tajik, Gana, & Danto, 1997), whilst performing normally on making superordinate-subordinate judgements about daily life event themes and component events once executive demands were controlled for (Wood, Tierney, Bidwell, & Grafman, 2005). Focal lesions of the ventromedial and medial frontopolar cortex rather than the dorsolateral frontal cortex were also associated with a reduced tendency to look into the long-term future (Fellows & Farah, 2005). Medial frontopolar neurodegeneration was further associated with selective impairments of complex socio-moral emotions such as guilt which require representing the long-term consequences of social behaviour (Moll et al., 2011) and this concorded with fMRI evidence on medial frontopolar activation for guilt (Basile et al., 2011, Kedia et al., 2008, Moll et al., 2007, Morey et al., 2012, Takahashi et al., 2004, Zahn et al., 2009b) relative to other complex negative emotions. Socially and emotionally-relevant event sequences, particularly those of personal relevance (Forbes & Grafman, 2010), were hypothesized to be represented in ventral frontal cortical areas (Moll et al., 2005, Wood and Grafman, 2003). To our knowledge, however, all previous neuropsychological investigations of sequential knowledge have used tasks that required active sequencing and it is thus impossible to clearly disentangle their requirement for executing a sequencing task from their requirement of knowing about the correct sequence.

FTLD is a heterogeneous disorder including clinical syndromes of semantic dementia (SD) and behavioural variant frontotemporal dementia (bvFTD) predominantly affecting anterior temporal and frontal, including frontopolar, cortices. Despite their anatomical heterogeneity, both variants display consistent and early inappropriate social behaviour (Bozeat et al., 2000a, Liu et al., 2004, McKhann et al., 2001, Snowden et al., 2001). The study of the cognitive neuropsychology of social behaviour in FTLD is therefore a crucial test of whether social knowledge is important for appropriate social behaviour. One possibility is that the atrophy to the frontal lobes in FTLD (Miller et al., 1991, Miller et al., 1997, Neary et al., 1993, Neary et al., 1998), is largely responsible for inappropriate social behaviour, as the posterior orbitofrontal cortex is regularly affected in SD as well (Liu et al., 2004, Mummery et al., 2000, Rosen et al., 2002). This is contradicted by the findings that ATL (Zamboni, Huey, Krueger, Nichelli, & Grafman, 2008) and ventromedial frontal cortex (including parts of the frontopolar cortex) are both independently associated with inappropriate social behaviour in FTLD (Liu et al., 2004). Thus, whilst it is largely undisputed that both frontal and ATL regions contribute to inappropriate social behaviour in FTLD (Sollberger et al., 2009), it is still unclear what their respective functional contributions are. One popular model derived from non-human animal conditioning experiments postulates disinhibited frontal suppression of subcortically-mediated behavioural urges (Brutkowski, 1965) resulting in “disinhibited” behaviour in frontal lesion patients. Underlying, the frontal suppression model is the assumption that frontal damage does not impair knowledge of the appropriateness of one's urges, only the ability to control them. There is contradictory evidence, however, on whether social knowledge is intact in patients with bvFTD who regularly demonstrate such damage (Grossman et al., 2010, Mendez, 2010, Mendez et al., 2005a, Mendez et al., 2005b). Furthermore, there has been no investigation of whether different patients with FTLD exhibit dissociable deficits between different forms of social knowledge, which could underpin shared social behavioural changes in patients with non-overlapping patterns of neurodegeneration.

So far, there has been no direct comparison of social sequential and conceptual knowledge in patients due to the lack of semantic tasks that probe sequential social knowledge. We designed such a novel task to probe knowledge of short- and long-term consequences of social behaviour and used this alongside our previously developed Social Concept Discrimination Task (Zahn, Moll, Iyengar et al., 2009) and a control task, the Visual Discrimination Task to investigate whether there are dissociable deficits in these knowledge forms that would support the hypothesis of their neuroanatomical separation (Moll et al., 2005). Specifically, we hypothesised that knowledge of social behaviour can be separated into three components which are neuroanatomically distinct and should therefore give rise to double dissociations at the single patient level (Shallice, 1990), such that we expected to find patients with 1) selective impairments on knowledge of long-term versus short-term consequences, 2) long-term versus social conceptual knowledge and the respective reverse dissociations. We further hypothesised that 3) patients with relatively reduced grey matter (GM) volumes in their frontopolar cortices versus right superior ATL would show poorer knowledge of long-term consequences relative to social conceptual knowledge and vice versa. Our approach is based on the cognitive neuropsychology method and the notion that impairments on neuropsychological tasks are due to multiple cognitive components, which is why we focus on the performance difference of a patient between closely matched task conditions that differ on a cognitive component to be probed (Shallice, 1990). These so called “dissociations” in performance point to a difference in performance between conditions due to differential impairment of the cognitive component varied between the conditions. Recent statistical approaches have been developed and are well established to formally probe single case dissociations (Crawford & Garthwaite, 2005).

## 2 Material and methods

### 2.1 Patients and controls

Patients were referred from old age psychiatry community teams, the Cerebral Function Unit (AG) at Salford Royal Hospital or seen in the South Manchester University Hospital Memory Clinic (RZ, AB). Four SD patients were assessed as part of a study led by MALR and described elsewhere (Hoffman, Jones, & Ralph, 2012). Patients were referred to MALR for study participation from the Research Institute for Care of the Elderly (RICE) institute in Bath after previously receiving a diagnosis of SD from a consultant neurologist (Prof. Roy Jones). Patients provided written informed consent for participation in accordance with a Multicentre Research Ethics Committee approved protocol and a South Manchester NHS Research Ethics Committee approved protocol which also allowed for carer assent-based inclusion if patients agreed to the study but lacked the capacity to consent. Participants were compensated for their time and travel.

All Manchester patients were clinically assessed by a senior old age psychiatrist (RZ) or senior neuropsychologist (MALR) and underwent extensive neuropsychological test examination as well as MRI in most cases or CT. General inclusion criteria for this study were strong right-handedness and English as first language. Diagnosis of FTLD (including those with features of bvFTD, or of SD, as well as mixed presentations) was based on clinical, neuropsychological criteria as well as visual inspection of MRI/CT scans and followed established consensus criteria [FTLD: Lund–Manchester criteria (Neary et al., 1998)] shown to have excellent neuropathological validity (Snowden et al., 2011) and which, contrary to more recent consensus criteria that strictly separate progressive aphasias and behavioural variants and were primarily developed by cognitive neurologists (Gorno-Tempini et al., 2011, Rascovsky et al., 2011), allow for mixed subtype presentations, which are common in our experience when detailed neuropsychiatric assessments are carried out.

SD patients had a history of at least two years of prominent impairment of communication without further clinically relevant symptoms. SD patients had fluent speech with comprehension and naming impairments as their lead symptom. As stipulated by the Lund–Manchester criteria (Neary et al., 1998), the lead symptoms in bvFTD patients were progressive dysexecutive or behavioural abnormalities with an early loss of insight as noted by caregivers. Early amnesia and visuo-spatial deficits were absent in the history of all FTLD patients. All FTLD patients showed abnormalities within frontotemporal areas upon visual inspection of MRI or CT scans.

24 patients with a suspected clinical diagnosis of FTLD were consecutively enrolled. 5 FTLD patients had to be excluded prior to the statistical analysis (*n* = 1 who struggled to complete the experimental tasks, *n* = 1 whose family withdrew assent, *n* = 3 where the assessment resulted in a different diagnosis: 1 affective disorder, 1 Alzheimer's dementia, 1 phenocopy syndrome) leading to a final FTLD sample of *n* = 19 (8 male, age: mean = 67.3 ± 8.3 years, education: mean = 12.3 ± 3 years; ±refers to standard-deviation throughout the text). FTLD patients had been clinically classified into SD (*n* = 5), SD with features of bvFTD (*n* = 2), bvFTD (*n* = 6), bvFTD with features of SD (*n* = 4) and mixed primary progressive aphasia (logopenic & SD) with secondary features of bvFTD (*n* = 1), as well as mixed primary progressive aphasia (nonfluent & SD) with secondary features of bvFTD (*n* = 1). The final sample of *n* = 19 control participants (10 male) was younger (age: mean = 60.7 ± 9.1; years *t* = −2.3, *p* = .03) and showed a trend towards being more highly educated (education: mean = 14.2 ± 3.2 years; *t* = 1.9, *p* = .06), which is why education- and age-effects were investigated as potential confounders in all analyses comparing FTLD versus control groups. Gender was matched between groups (Contingency Coefficient = .11, *p* = .52). 15 participants from each group were included in the MRI part of the study with gender (Contingency Coefficient = .13, *p* = .46) and education (*t* = 1.5, *p* = .14) matched, but a trend towards healthy control participants being younger (*t* = −1.8, *p* = .08). 4 patients were not able to undergo research MRI, because they were assessed in Bath.

All 25 healthy volunteers had no history of psychiatric, neurological or relevant medical disorders as assessed by a clinical history with specific probe questions (one had been excluded before on screening because of a history of hypothyroidism and one because of epilepsy). Of the 25 control participants, three had to be excluded because of abnormal results on the Addenbrooke's Cognitive Examination-R, and three were excluded because of MRI abnormalities (excessive atrophy: 1, marked cerebrovascular small vessel disease: 1, arachnoid cyst: 1) resulting in a final sample of *n* = 19 for the neuropsychological part of the study, 4 were excluded from the MRI part of the study because of MRI contraindications leading to a final sample of *n* = 15 for the MRI study.

### 2.2 Neuropsychological test examination

Core background neuropsychological tests included the Addenbrooke's Cognitive Examination [ACE-R, version A, 2005 (Mioshi, Dawson, Mitchell, Arnold, & Hodges, 2006)], and the Cambridge 64 item Semantic Battery including Naming, and Word-to-Picture Matching (Bozeat, Lambon Ralph, Patterson, Garrard, & Hodges, 2000).

Participants completed the Visual Discrimination Task, Social Concept Discrimination Task, and the Consequences of Social Action Task without prior training as self-paced tasks using the presentation software E-Prime, http://www.pstnet.com/eprime.cfm, version 1.1. They were designed in analogy to the Pyramids and Palm Trees Test (Howard & Patterson, 1992) in which a probe item was presented at the top of a screen and participants had to decide which of two stimuli at the bottom was more related to the probe. For all tasks, patients and control participants were asked to point to their chosen response on the screen and the experimenter operated the keys to register the response. The stimuli were always presented in a random order and the position of the target and distracter was counterbalanced across trials. The experimenter read the probe word and pointed to each of the stimuli at the bottom in turn, starting with the one on the left, and then the one on the right. Stimuli were presented in a white font on a black background.

**Fig. 1:**
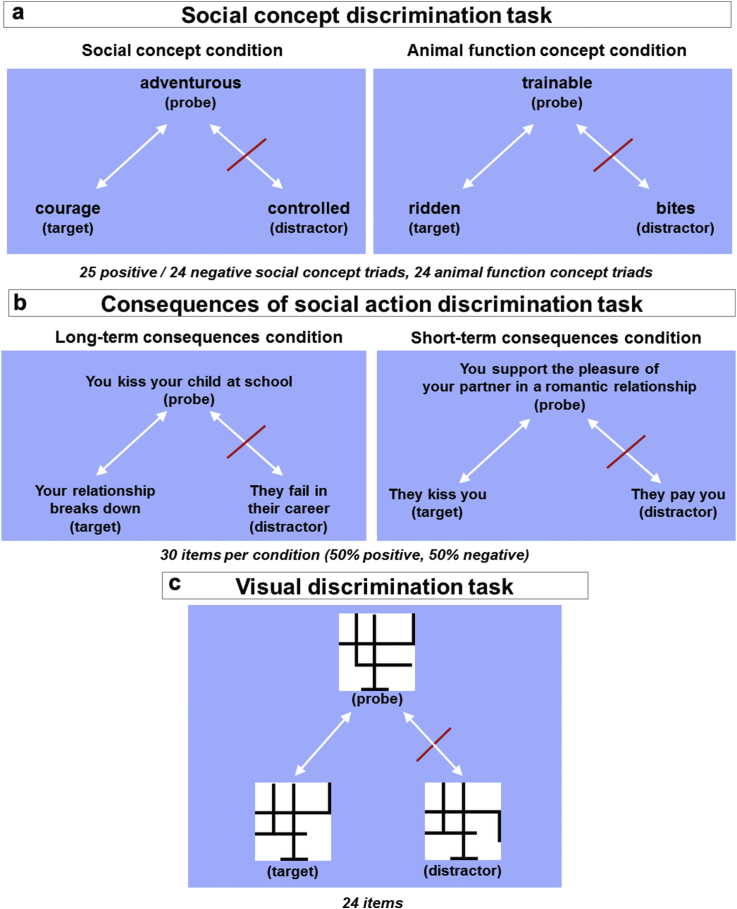
Overall design of (a) Social Concept Discrimination Task (Zahn, Moll, Iyengar et al., 2009), (b) consequences of Social Action Discrimination Task, (c) Visual Discrimination Task.

The Visual Discrimination Task is a non-verbal control task (Fig. 1c), created by SG and was administered to control for the task demands of the Social Concept Discrimination Task and the Consequences of Social Action Task. It is comprised of 2D line drawings of black and white figures that are of no resemblance to real objects (Gauthier, James, Curby, & Tarr, 2003); stimulus images courtesy of Michael J. Tarr, Center for the Neural Basis of Cognition and Department of Psychology, Carnegie Mellon University, http://www.tarrlab.org/. The original line drawings were slightly modified to produce 24 triads whereby there is a probe figure, a target figure that is similar (not identical) to the probe (only one difference in line arrangements between the two) and a distracter figure that is different to both (three differences in lines between distracter and probe, and between distracter and target). A target that was similar rather than the same as the probe was used so that the task reflected the demands of the Social Concept Discrimination Task. The participant was asked to point to the “figure which is most related to the figure at the top”.

For the Social Concept Discrimination Task [previously described by Zahn, Moll, Iyengar et al. (2009), Fig. 1a] target (e.g., “courage”) and distracter (e.g., “controlled”) concepts were chosen from the same category as the probe concepts (e.g., social concept probe: “adventurous”; animal function concept probe: “trainable”, target: “ridden”, distracter: “bites”) and had been used in our previous fMRI studies (Zahn et al., 2007, Zahn et al., 2009b). Participants were presented with six training triads followed by 73 stimulus items presented in a random order (25 positive social concept triads, 24 negative social concept triads, 24 animal function concept triads). The full description of matching of relevant psycholinguistic variables and data from *n* = 30 controls, *n* = 29 FTD and *n* = 18 corticobasal degeneration patients are available in Zahn, Moll, Iyengar et al. (2009). Stimulus items were previously established to have 80% response agreement in a normative study (Zahn, Moll, Iyengar et al., 2009). Participants were asked to point to the word which was most related to the word at the top (experimenter points to the probe at the top of the screen).

The Consequences of Social Action Task, created by SG, was then administered (for details of the design of the task, please see Supplementary Materials and Fig. 1b). This task was comprised of 60 triads with a probe social behaviour, and two alternative consequences, a target consequence that was more related to the behaviour, and a less related distracter consequence (30 long-term consequences: 15 positive and 15 negative, 30 short-term consequences: 15 positive, 15 negative). In any one trial, both of the consequences (target and distracter) were either long-term or short-term. Participants were asked to point to the “consequence which was most related to the social behaviour”, and the experimenter pointed to the statements whilst explaining the instructions. Example of short-term consequences (Probe: “You punish an employee at work”, Target: “They verbally attack you”, Distracter: “They violently attack you”). Example of long-term consequences (Probe: “You ignore your employers' requests at work”, Target: “Your standard of living decreases”, Distracter: “You are violently attacked”). All experimental tasks are available at: http://www.translational-cognitive-neuroscience.org/start/test-materials or from the authors.

### 2.3 Statistical analysis of experimental tasks

We tested for strong dissociations between two impaired scores on two task conditions (% correct), using a modified paired samples *t*-test and the Revised Standard Difference Test (RSDT) of Crawford and Garthwaite (2005) implemented in the software created by the authors (http://www.abdn.ac.uk/∼psy086/dept/SingleCaseMethodology.htm). The RSDT has been shown to adequately control for error rates even in small samples (Crawford & Garthwaite, 2005) and is a well-established method for probing single case dissociations. The mean and standard deviation for each test/condition from the control group was entered, the correlation between scores on these tests conditions/tests, together with the sample size of the control group and the patients scores on the two tests. Dissociations were considered at an individual threshold of *p* < .0026 (corresponding to an approximate Bonferroni-corrected *p* < .05 across 19 single patient cases) for long-term versus short-term consequences and social concept versus long-term consequences conditions.

The only potential variables to be controlled for in patients with selective impairments on long-term versus short-term consequences that differed between the conditions were target median familiarity and median likelihood (lower in the long-term consequences condition). This was achieved by computing individual binary logistic regression models (SPSS 15.0) for each subject in the FTLD group in which a significant dissociation between long-term and short-term consequences conditions had occurred. Familiarity and likelihood per stimulus were predictor covariates in separate models and correct/incorrect response the categorical outcome in both models (no constant modelled).

### 2.4 Image acquisition

All images were acquired with a Philips Achieva 3T scanner using an 8-channel head-coil. The T1-weighted sequence was a high-resolution 3D MPRAGE, inversion time 1150 msec, 256 × 164 matrix, 128 slices, voxel size = 1 mm^3^, TE = 3.8 msec, TR 9.4 msec, flip angle 80. T2-weighted images were acquired using a turbospin-echo sequence with SENSE factor 2. Matrix size was 512 × 408, 44 slices, voxel size .26 mm × 0.26 mm × 3.30 mm, TE 80 msec, TR3s, flip angle 900. This sequence was used to exclude participants with marked cerebrovascular, or other non-degenerative changes.

### 2.5 Image analysis

Imaging data were analyzed using statistical parametric mapping (SPM8, http://www.fil.ion.ucl.ac.uk/spm/software/spm8). Images were inspected for artefacts (motion, high level of inhomogeneities) before and after normalization. T1-images were normalized and segmented into GM, white matter (WM) and cerebrospinal fluid (CSF) using the new features of the VBM8 toolbox, version 359 (http://dbm.neuro.uni-jena.de/vbm): segmentation without tissue priors, labelling voxels according to their tissue types using partial volume estimation (PVE), de-noising with non-local means filter and integration of DARTEL normalization (Ashburner, 2007). After pre-processing, images (1.5 × 1.5 × 1.5 mm^3^ voxel size) were smoothed with a Gaussian kernel of FWHM = 12 mm. The “non-linear modulation only” option (i.e., with no affine component) was selected to create volumetric GM and WM partitions. This option is recommended to obtain relative volume after correcting for differences in brain size and replaces earlier methods of using total intracranial volume or total GM + WM (http://dbm.neuro.uni-jena.de/vbm/segmentation/modulation/) as nuisance covariates. Thorough clean-up was selected as recommended for atrophied brains. A .2 absolute threshold masking was used to select voxels for the subsequent statistical analysis. We extracted the voxel grey matter volumes in our two a priori regions of interest (ROIs): frontopolar cortex (Brodmann Area [BA] 10; MNI: −2, 66, 20) and right superior ATL (MNI: 58, 0, −12) and our two control regions: subgenual cingulate cortex (MNI: −4, 23, −5) and left superior ATL (mirror region of the right) using 6 mm radius spheres around centre coordinates from our previous paper revealing functional connectivity changes in this network in major depressive disorder which is associated with overgeneralized interpretations of social behaviour (Green, Ralph, Moll, Deakin, & Zahn, 2012) using the Marsbar toolbox (Brett, Jean-Luc Anton, Valabregue, & Poline, 2002). The subgenual cingulate was chosen as a control region because it is part of a posterior ventromedial frontal region that has been associated with impaired judgments of acceptability of social rule violations in patients with FTLD (Grossman et al., 2010) and is functionally connected with our right superior ATL region whilst evaluating social behaviour (Green et al., 2010).

## 3 Results

### 3.1 Group comparisons

Group comparisons of our patients with healthy control participants (Table 1) showed that general semantic and social conceptual impairments were consistently observed in our FTLD group, whereas more variability existed in performance on the sequential social knowledge task. As expected, almost all patients performed normally on the Visual Discrimination Task.

**Table 1:** Group comparisons on core neuropsychological tests.

	Control mean ± standard deviation	Control *n*	FTLD mean ± standard deviation	FTLD *n*	*p*-Value	FTLD ratio number of cases impaired/total
Addenbrooke's cognitive examination-revised score	96.5 ± 3.2	19	63.9 ± 15.35	19	<.0001*	18/19
Cambridge Semantic Test Battery – naming (64 items) [number correct]	63.5 ± .61	19	40.3 ± 17.21	17	<.0001*	17/17
Cambridge Semantic Test Battery – word to picture matching (64 items) [number correct]	63.6 ± .60	19	49.8 ± 13.34	18	<.0001*	18/18
Visual discrimination [% correct]	90.6 ± 6.77	19	92.8 ± 7.05	19	.335^n.s.^	1/19
Animal function concept discrimination [% correct]	93.6 ± 5.07	19	80.9 ± 12.98	19	.001*	12/19
Social Concept Discrimination [% correct]	89.8 ± 4.03	19	65.7 ± 14.51	19	<.0001*	16/19
Short-term consequences discrimination [% correct]	97.4 ± 3.61	19	90.3 ± 8.75	19	.004*	10/19
Long-term consequences discrimination [% correct]	87.2 ± 3.89	19	75.4 ± 11.82	19	<.0001*	9/19

The number of patients with impairments (<healthy control mean – 2 standard deviations). Naming and word-to-picture naming tasks were taken from the Cambridge Semantic Test Battery (64 items). * marks significance at 2-tailed *p* < .05. n.s. marks non-significance at 2-tailed *p* = .05.

### 3.2 Dissociations at the individual level

**Fig. 2:**
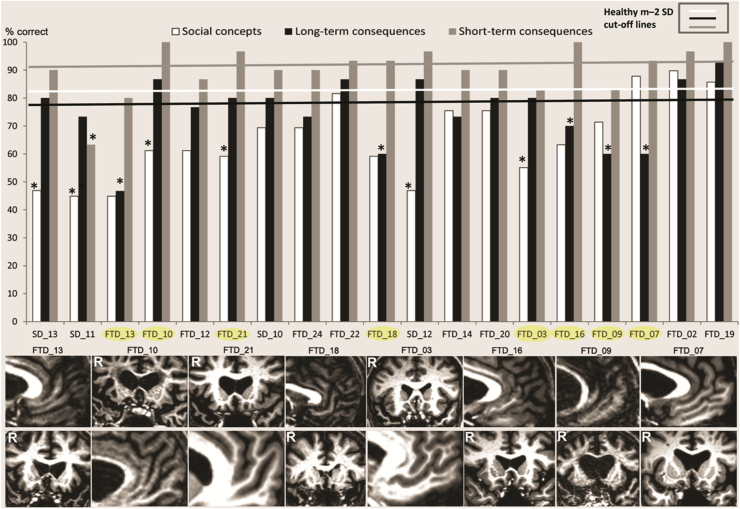
Performance on the Social Concept (white), Long-term Consequences (black), and Short-term Consequences (grey) task conditions are displayed for each patient (% correct). Patients are ordered according to their Addenbrooke's Cognitive Examination-R scores (left for lowest to right for highest scores). White, black, and grey lines represent healthy control means – 2 standard deviation cut-off values for each task. Using a well-established method (Crawford & Garthwaite, 2005), significant dissociations at *p* < .0026 (corresponding to an approximate Bonferroni-corrected *p* < .05 across 19 single patient cases) for Long-term versus Short-term Consequences and Social Concept versus Long-term Consequences conditions were marked with an asterisk. There was one patient with a selective impairment on Long-term Consequences versus Social Concepts (FTD_7). Another patient displayed a selective impairment on Short-term versus Long-term Consequences (SD_11). The upper row of T1-weighted MRI scans displays coronal sections through the anterior temporal lobes for 3 patients showing a Social Concept-selective impairment relative to Long-term Consequences (FTD_10, FTD_21, FTD_3), no MRI scans were available for additional 3 patients showing this dissociation (SD_13, SD_11, SD_12). The upper row also shows mid-sagittal slices through the frontopolar and ventromedial frontal cortices of all the 5 patients exhibiting a selective impairment for Long-term versus Short-term Consequences (FTD_13, FTD_18, FTD_16, FTD_09, FTD_07). The lower row displays slices to depict the 2nd region of interest for comparison (frontopolar for patients with Social Concept-selective and anterior temporal for patients with Long-term Consequences-selective impairments).

As predicted, we observed double dissociations between our three social knowledge task conditions of interest. One patient exhibited a selective impairment on long-term consequences versus social concepts (FTD_7). He was also among five patients displaying selective impairments on long-term consequences relative to short-term consequences (Fig. 2; FTD_07, FTD_09, FTD_13, FTD_16, FTD_18). Another patient displayed a selective impairment on short-term versus long-term consequences (SD_11). In contrast, six patients showed selective impairments on social concepts relative to long-term consequences (FTD_3, FTD_10, FTD_21, SD_11, SD_12, SD_13).

All five patients with selective impairments on long-term versus short-term consequences were further examined using individual logistic regression models to determine the potential confounding influence of lower target consequence likelihood and familiarity in the long-term relative to the short-term consequences condition. Target consequence familiarity had no effect on task performance in any patient (*p* > .07, Wald < 3.4), and likelihood significantly affected performance only in one case such that target consequences with higher rated likelihood were more often correctly selected (FTD_16: *B* = .036, Standard error = .018, Wald = 3.87, df = 1, *p* = .05). The condition effects on performance (long-term < short-term consequences) disappeared when correcting for effects of likelihood as a covariate in this case.

### 3.3 Neuroanatomy of performance differences between long-term consequences and social concepts

There were no significant effects of age or education on the differences in performance between long-term consequences relative to social concepts (Control group, *n* = 19, age: rho = .10, *p* = .68, education: rho = −.28, *p* = .24; FTLD group, *n* = 19, age: rho = .06, *p* = .79, education: rho = −.24, *p* = .33) or relative to short-term consequences (Control group, *n* = 19, age: rho = .27, *p* = .27, education: rho = −.13, *p* = .59; FTLD group, *n* = 19, age: rho = .19, *p* = .43, education: rho = .05, *p* = .83) in either group.

**Fig. 3:**
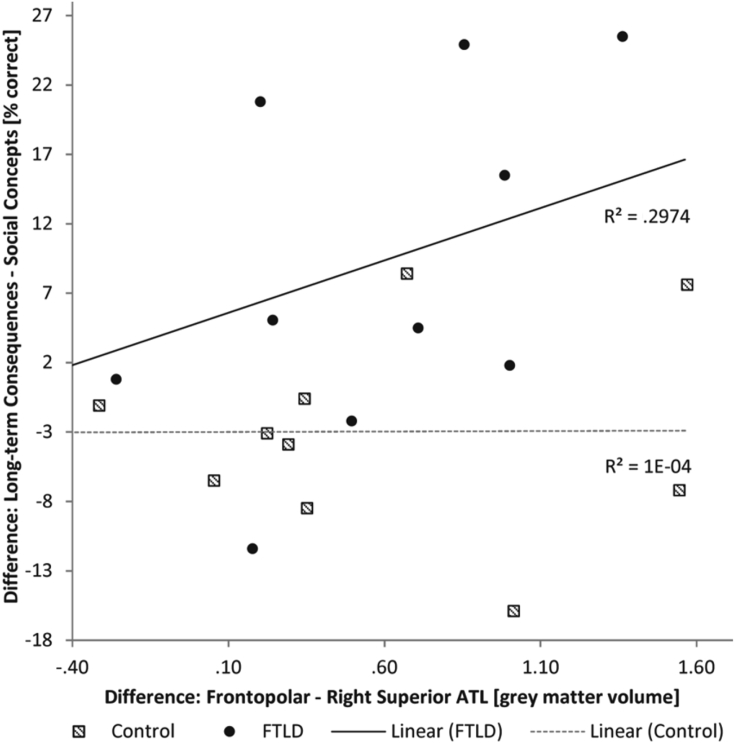
Difference scores for performance on the Long-term Consequences versus Social Concept task conditions [% correct] were plotted against difference scores in frontopolar cortex (i.e., BA10) versus right superior ATL grey matter volumes in *n* = 15 FTLD patients and *n* = 15 healthy control participants. There was a positive association between both difference scores in the FTLD group such that lower frontopolar relative to ATL volume was associated with poorer performance on Long-term Consequences relative to Social Concepts and vice versa. The control group showed no such relationship between neuropsychological and brain region difference scores.

As hypothesised, difference scores for performance on the long-term consequences versus social concept task conditions were positively associated with difference scores in frontopolar cortex (i.e., BA10) versus right superior ATL grey matter volumes in the FTLD group (*F*[14,1] = 5.50, *p* = .04, *R*^2^ = .30, Fig. 3). Lower frontopolar relative to ATL volume was associated with poorer performance on long-term consequences relative to social concepts and vice versa. This was also the case when correcting for age (*t* = 2.35, partial beta = .56, *p* = .04 for the effect of interest with no effect of age: *t* = −.65, partial beta = −.12, *p* = .53). The control group showed no such relationship between neuropsychological and brain region difference scores (*F*[14,1] = .001, *p* = .97, *R*^2^ < .0001). As expected there was also no association when using a difference score of volume in our control regions subgenual cingulate – left superior ATL (*F*[14,1] = .60, *p* = .45, *R*^2^ = .04). In a supporting analysis we also modelled the frontopolar versus right superior ATL difference score together with the control region difference score and demonstrated that the association of our regions of interest with our neuropsychological measure of interest remains significant even when directly adjusting for the effects of the control regions (partial eta squared = .41, *t* = 2.9, *p* = .01). There was no association between our neuropsychological measure of interest and grey matter volumes when using a difference score of volume in the frontopolar cortex – left superior ATL (*F*[14,1] = .63, *p* = .44) showing that no similar relationship exists for the left superior ATL.

All values on the difference scores used were within 2.5 standard deviations of the respective group mean suggesting that outlying values were unlikely to influence the results. This was further corroborated by using additional non-parametric (i.e., rank-rather than normal distribution-based) statistics. This confirmed the positive correlation of long-term consequences versus social concept task difference scores with difference scores in frontopolar cortex versus right superior ATL grey matter volumes in the FTLD group (Spearman rho = .51, *p* = .05).

## 4 Discussion

We confirmed our hypotheses that knowledge of long-term consequences of social behaviour can be dissociated from both the knowledge of short-term consequences and social concepts. This finding indicates at least partly distinct neuroanatomical representations. We further confirmed our hypothesis that frontopolar cortical relative to right superior ATL grey matter volume loss is associated with poorer performance on long-term consequences relative to social concepts and vice versa. This provides the first direct association of neuropsychological probes of sequential knowledge with frontopolar cortical volumes.

These findings are in keeping with the hypothesis that semantic (i.e., non-episodic) knowledge of social behaviour can be divided into a conceptual component within the ATL and a non-conceptual component in the frontal cortex (Zahn, de Oliveira-Souza, & Moll, 2011). The high frequency of impairments on knowledge of long-term consequences in our FTLD group is also in keeping with findings from glucose positron emission tomography (PET) showing that ventromedial frontopolar areas are most consistently impaired in bvFTD (Salmon et al., 2003).

Our finding of impairments of long-term sequential social knowledge in about half of our FTLD patients including patients with bvFTD contradicts the conclusions drawn from previous studies assessing social knowledge in bvFTD with clinical interviews. Mendez, Chen et al. (2005) did not find social knowledge in their bvFTD sample to be impaired and stated that all 16 patients had knowledge of the outcomes of their behaviours and “could describe the potential consequences of their actions, but 12 did not feel ‘very concerned’ about them. Nine patients minimized the impact of their behavior, but none gave more extensive rationalization”. Another study by Mendez and Shapira (2009) probed FTD patients' social knowledge by asking them how right or wrong certain actions are (e.g., “take the last seat on a crowded bus”), and revealed no impairments in knowledge of these social conventions. A possible explanation for the discrepancy with our finding is that the knowledge of behaviour probed in these studies did not require consideration of long-term consequences because they were overlearned social ‘rules’ or may not result in long-term consequences per se.

Interestingly, in contrast to Mendez and Shapira, 2009, Grossman et al., 2010 demonstrated that FTLD patients more readily endorse statements asking for the social acceptability of social violations than healthy control participants, therefore demonstrating impaired social knowledge. The items used in this study included the violation of a social convention (e.g., “running a red light”) as well as additional contextual detail, (e.g., “You run a red light at 2 am in the morning”) and so may have more readily demanded sequential knowledge due to the variability of different consequences that could occur. For example, whilst running a red light is an over-learned driving violation, the consequence that may arise is very different if it occurs at 2 am in the morning (e.g., “no one else will be around” ⇒ “I might not get caught” ⇒ “I won't cause an accident”), rather than in the middle of the day (e.g., “lots of people around” ⇒ “someone could get hurt” ⇒ “I might get caught” ⇒ “I could get into trouble with the law”). This could explain differences between the results of (Mendez & Shapira, 2009) and Grossman et al. (2010), where the authors hypothesised that the impairments of FTLD patients could arise from their inability to anticipate the negative consequences of their behaviour; a hypothesis which is directly supported by our results.

Our results also need discussing in the context of studies in patients with non-neurodegenerative ventral frontal lobe lesions which have consistently demonstrated that the integrity of this region is critical for normal moral development and social conduct (Anderson et al., 1999, Anderson et al., 2006, Blair and Cipolotti, 2000, Eslinger and Damasio, 1985). EVR developed impairments in social behaviour as a result of removal of a meningeoma in the ventral frontal lobes (Eslinger and Damasio, 1985, Saver and Damasio, 1991). In contrast to our findings, however, his performance on a task probing his knowledge of the consequences of social behaviours was intact (Saver & Damasio, 1991). One possible explanation for this discrepancy is that the employed test may have relied more heavily on probing short-rather than long-term consequences and may have also relied on overlearned associative knowledge which may be more redundantly stored. Future studies, comparing performance of patients with ventral and frontopolar brain lesions on our test as well as other tests of social knowledge are required to fully account for the discrepancies.

The relationship between clinical diagnosis and profile of social knowledge impairment was examined in a supporting analysis (Supplementary Table 4) which was in keeping with our main analysis such that patients with suspected primary involvement of the ATLs (i.e., those with primary SD or primary atrophy of the ATLs) performed more poorly on social concepts relative to long-term consequences and showed lower ATL relative to frontopolar volume. As expected, the reverse pattern arose for patients whose neurodegeneration was suspected to have started outside the ATL on the basis of their clinical presentation and/or neuroimaging inspection (primary bvFTD or no ATL atrophy). This analysis was not the main focus of our paper, because of the uneven distribution of clinical groups with only a small number of non-ATL patients. The higher proportion of ATL patients was also reflected in the overall pattern of grey matter volume loss which consistently involved anterior temporal, but not frontopolar brain regions (Supplementary Fig. 3 and Supplementary Table 5).

In further supporting analyses (Supplementary Tables 2 and 3), we showed that performance on general semantic tests and the verbal subtests of the ACE-R in our patients was associated with performance on social concepts as we would have predicted, but interestingly to similar degrees also between general semantic tests and short-term consequences, but not the long-term consequences task. We know that general semantic abilities are required for all tasks, but it appears that differences in performance between patients on long-term consequences were driven by additional requirements not captured by standard semantic tests. In contrast, part of the differences in performance on short-term consequences could have been driven by general semantic abilities. Alternatively, general semantic abilities could have been associated with performance on short-term consequences, because patients with SD were shown to exhibit neurodegeneration in posterior ventral frontal areas (Liu et al., 2004, Mummery et al., 2000, Rosen et al., 2002) hypothesised to represent short-term (i.e., less complex) social consequences/outcomes (Moll et al., 2005, Wood and Grafman, 2003). This highlights the importance of restricting one's interpretation to dissociations between tasks rather than overall task performance as we have done throughout this paper.

On a more cautionary note, we did not have the opportunity to gather glucose PET data in our patients, known to be more sensitive to early neurodegenerative changes, especially in the frontal cortex in FTLD (Salmon et al., 2003) compared to T1-weighted structural MRI (Hodges, 2001). Future replication of our association of relatively pronounced frontopolar damage with relatively pronounced impairments on long-term consequences are therefore needed using a more sensitive neuroimaging probe of frontal integrity. Ideally, future studies would aim for a larger subgroup of patients with frontopolar neurodegeneration who do not exhibit ATL pathology and a larger overall sample size to provide more reliable estimates of effect size. This will be challenging, however, because at a group level, patients with bvFTD also demonstrated ATL involvement (Salmon et al., 2003), but prospective studies in families of patients with FTLD may provide such an opportunity. The battery of experimental tests to probe social knowledge in the current study is well suited for further investigations, because the overall task demands were similar between the different conditions (two forced choices matched to a probe) and the verbal working memory load between short-term and long-term consequences task conditions was matched.

## 5 Conclusions

Taken together, our data support the hypothesis that knowledge of long-term consequences of social behaviour is dissociable from knowledge of its short-term consequences, as well as of social concepts. These cognitive neuropsychological dissociations support separable neuroanatomical representations of these different components of social knowledge. Furthermore, we found support for the hypothesis that frontopolar cortical volume loss is selectively associated with impoverished knowledge of the long-term consequences of social behaviour, whilst confirming our previously established association of disrupted social conceptual knowledge with right superior ATL neurodegeneration. Future studies are needed to confirm the proposed role of the frontopolar cortex in representing long-term consequences of social behaviour. Our results suggest that rather than being unable to suppress urges to behave inappropriately, FTLD patients often lose the knowledge of what appropriate social behaviour is and can therefore not be expected to behave accordingly. Patients losing knowledge of the long-term consequences of behaviour can be expected to act seemingly more “impulsively”, without having to postulate a deficit in their ability to suppress urges and such claims should be used more cautiously.
