# Supplementary material for: Frontotemporal lobar degeneration and social behaviour: Dissociation between the knowledge of its consequences and its conceptual meaning
Source: Cortex. 2017 Aug;93:107–18. doi: 10.1016/j.cortex.2017.05.009 (PMC5542070; doi:10.1016/j.cortex.2017.05.009)
Supplement: Supplementary file 1 [file mmc1.docx]

**Supplementary Materials**

**Frontotemporal lobar degeneration and social behaviour: dissociation between the knowledge of its consequences and its conceptual meaning**

Roland Zahn^1,2,*^, Sophie Green^2^, Helen Beaumont^2,3^, Alistair Burns^4^, Jorge Moll^5^, Diana Caine^2,6^,

Alexander Gerhard^4^, Paul Hoffman^2^, Benjamin Shaw^4^, Jordan Grafman^7^, Matthew A. Lambon Ralph^2^

*^1^ Institute of Psychiatry, Psychology & Neuroscience, Department of Psychological Medicine, King’s College London, London, SE5 8AZ, UK*

*^2^ Neuroscience and Aphasia Research Unit, Division of Neuroscience and Experimental Psychology, The University of Manchester, Manchester, UK*

*^3^ Department of Neurology, Washington University School of Medicine, St. Louis, Missouri, USA*

^4^ *Division of Neuroscience and Experimental Psychology, The University of Manchester, Manchester, UK*

^5^ *Cognitive and Behavioral Neuroscience Unit, LABS-D’Or Hospital Network, Rio de Janeiro, RJ, Brazil*

^6^ *National Hospital for Neurology & Neurosurgery, Queen Square, London, UK.*

^7^ *Rehabilitation Institute of Chicago, Chicago, Illinois & Department of Physical Medicine and Rehabilitation, Northwestern University, Chicago, Illinois, USA*

** To whom correspondence should be addressed:*

Dr. Roland Zahn (see address above)

E-mail: roland.zahn@kcl.ac.uk

Phone: 0044-(0)20 7848 0348

Fax: 0044-(0)20 7848 0298

## Supplementary Methods

***Consequences of Social Action Task Design***

We created probe items which were descriptions of social behaviours, and target/distracter items which described consequences of social behaviours, using normative data from two pre-studies. Social behaviours included a specific agent and recipient and the overall context was specified (particular situation). Target/distracter items were either short- or long-term consequences.

*1.1.1 Social behaviour probe generation*

The first normative study using the “Social Scenario Generation Task” was carried out at the National Institutes of Health, Bethesda, USA and has been described elsewhere([Green et al., 2013](#_ENREF_3)). In brief, participants were asked to provide social behaviours that are examples of social concepts (e.g. an example of a behaviour that is “stingy”, or “tactless”). Two independent raters obtained good inter-rater agreement for categorizing the responses into categories for recipient role, primary social action, and sequential context in order to group similar social behaviours together. These categories were then synthetically re-combined to create the social behaviour probes for the development of our novel task here.

We identified the two or three most frequently co-occurring categories for recipient role (e.g. “friend”), primary social action (e.g. “helping”) and sequential context (e.g. “at work”, “at school”) in order to create social behaviours that occurred between two people in particular situations. The category labels for the primary social action derived from the pre-studies were used without changes (e.g., “helping”, “giving a gift”, “ignoring requests”) as the social behaviour probes. The only exception was for the category of ‘inferior role’ for recipient/agent roles (e.g. an “amateur musician” is inferior to a “professional musician” when “playing a piece at a recital”); in this case another recipient was used.

Highly related categories were collapsed for stimulus creation (e.g. “verbally attacking”/”criticizing”/”accusing”), and the most relevant or appropriate term was used for each social behaviour probe, given the recipient role and the sequential context, in order to create variation between the stimuli. The median pleasantness and unpleasantness of the stimuli were determined to create social behaviour probes that were either positive or negative by aggregating the median of rated pleasantness and unpleasantness over the co-occurring primary social action, recipient role and sequential context categories. The created social behaviour descriptions were then presented as probe stimuli in the second normative study that was used to generate data about the anticipated consequences of social knowledge.

*1.1.2 Consequences of Social Action Task Normative Study*

The task was comprised of 15 pleasant social behaviour probes and 15 unpleasant social behaviour probes. The order of presentation of positive vs. negative stimuli was randomized across participants. They were entered into Excel Microsoft Visual Basic Editor and presented to 22 healthy participants (15 female, age: 24.0±8.4 years, education: 17.3±3.4 years) who gave written informed consent at the School of Psychological Sciences at the University of Manchester, where research ethics approval had been obtained. Participants were first required to complete a free association task using the social behaviour probes. Participants had to write down the first thing that enters their mind when reading the description of social behaviour.

Participants then completed the short-term consequences task (Supplementary Figures 1 and 2). They gave a detailed example of the general behaviour described in the stimulus, and then provided a short-term, immediate consequence of this specific behaviour, rated the pleasantness/unpleasantness of the short-term consequences (-4/+4 bipolar scale) as well as the probability of this as a consequence of the social behaviour described in the probe sentence (an estimate between 0 – 100 %) and how familiar they are with the consequence (1=not at all familiar – 7= very familiar).

They then completed the long-term consequences task, providing a chain of events that occurred in sequence following from the short-term consequence and resulting in a long-term, ultimate consequence. The long-term consequence had to be at least 5 years into the future. They then rated the pleasantness/unpleasantness of the long-term consequences as well as the probability of the consequences occurring and how familiar they are with them (all measures on the same scales as for the short-term consequence).

*1.2.3 Creating consequence categories (targets and distracters)*

We categorized participants’ responses based on features of the consequence generated using categories which were similar enough so that very different behaviours were kept separate but broad enough so that similar consequences could be grouped. This was done for both short-term and long-term consequences. Categories were created for the primary social action (e.g. in the short-term consequence condition: “violently attacks”, “verbally attacks”, “punishes”, “thanks”, “rejects”, “gets found out”, “donates possessions”; in the long-term consequence condition: “develops health problems”, “gets into trouble with the law/sent to prison”, “succeeding in their career”, “failing in their career”, ”has children”), for the agent and recipient role of the behaviour (e.g. “child - parent”, “spouse”, “employer-employee”), and who it is that the consequences would affect (self, other).

*1.2.4 Categorization of consequence data from normative study*

Occasionally data had to be recoded for participants who had not entered the consequence into the correct pre-defined field. On rare occasions for the long-term consequences, responses were too abstract or broad to be concretely defined as a consequence (e.g. “live a happy life”) and so the consequence provided before as one of the ‘steps’ leading to the long-term consequence were taken, or the ‘gist’ of the statement had to be inferred from previous information provided by the rest of the data for that item from that participant. Occasionally, participants had not provided a long-term consequence that was far enough into the future, and instead had replicated the short-term consequence that they had provided. In this case, their long-term consequence result was not used. Sometimes, the ultimate long-term consequence that was provided was of a different valence to the rest of the ‘steps’ provided by the participant, and in such cases the consequence step before this was used. In cases where participants provided a chain of consequences that were completely of the opposite valence to the probe, their data were excluded for that item.

*1.2.5 Construction of stimuli for the final patient task*

The probe labels were used as derived from the pre-studies, however the agent roles of the social behaviour were transformed into “You”, with stimuli presented as e.g., “You kiss your colleague at work”. A number of criteria were generated and followed when producing the stimuli:

1. The target consequence was chosen based on its frequency of its association with the probe category in our normative data. In most cases, the most frequently associated category was selected as the target. In these cases, the distracter was a consequence category that either occurred once or not at all as associated with that particular social behaviour probe. In a few cases, there was a variety of consequence categories associated with a given social behaviour probe. In these cases the target was associated with the probe at least twice, and the distracter had to be selected from responses generated to an unrelated social behaviour probe.
2. The target and distracter were produced so that they were equally conceptually related to the stimulus item. For example, take the probe *“You achieve at work”*, and consider the possible alternatives- the target could be *“You get a promotion”* and the distracter could be *“You develop health problems”*. Here, “*work”* is conceptually more related to “*promotion”* than “*health problems”*. Thus the distracter would not have been chosen because it would have allowed a correct response based on conceptual rather than sequential relationships. To make distracters more or equally related to the probes than targets, sometimes the distracter would be formulated by negating a previously positive social behaviour.
3. We did not introduce a third party into the stimuli, because this could have increased task demands for the FTLD group.
4. The distracter and target were not consequences of one another, for example the target *“You fail in your career”*, would not have been used following distracter *“Your standard of living decreases”.*
5. Care was taken, not to allow the target and distracter being examples of one another (i.e. a superordinate-subordinate relationship, e.g. *“You are rewarded”* and *“You are promoted”*).

**Supplementary Figures**

Supplementary Figure 1**.** Short-term consequences task pre-study. Participants completed the fields to provide a dataset of consequences which are perceived to occur after particular social behaviours to enable construction of short- and long-term consequence target and distracters for the *Consequences of Social Action Task.*


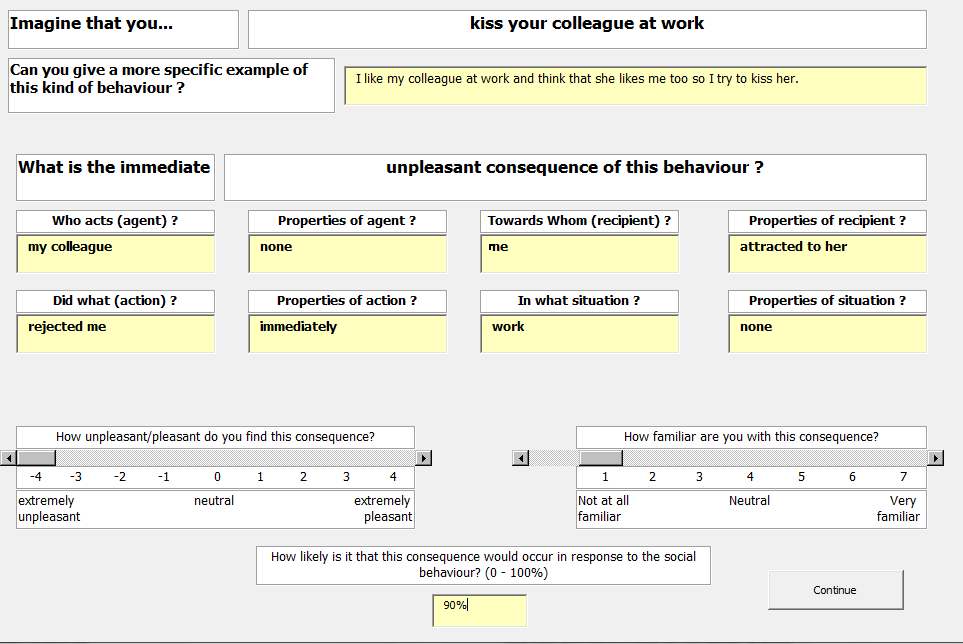

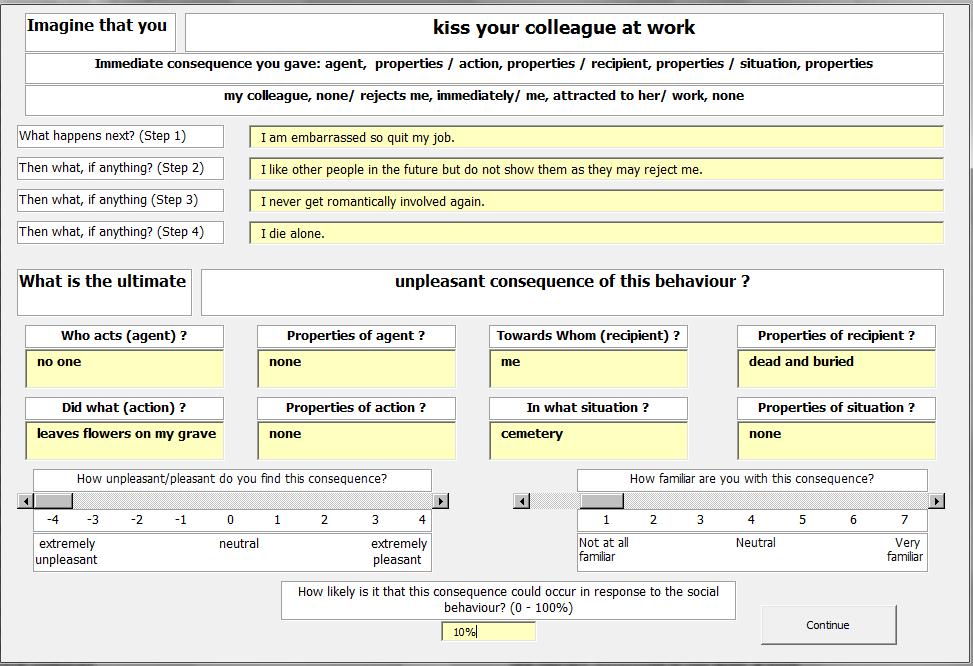


Supplementary Figure 2. Long-term consequences task pre-study, required for generation of consequence target and distracters for *Consequences of Social Action Task.*

**
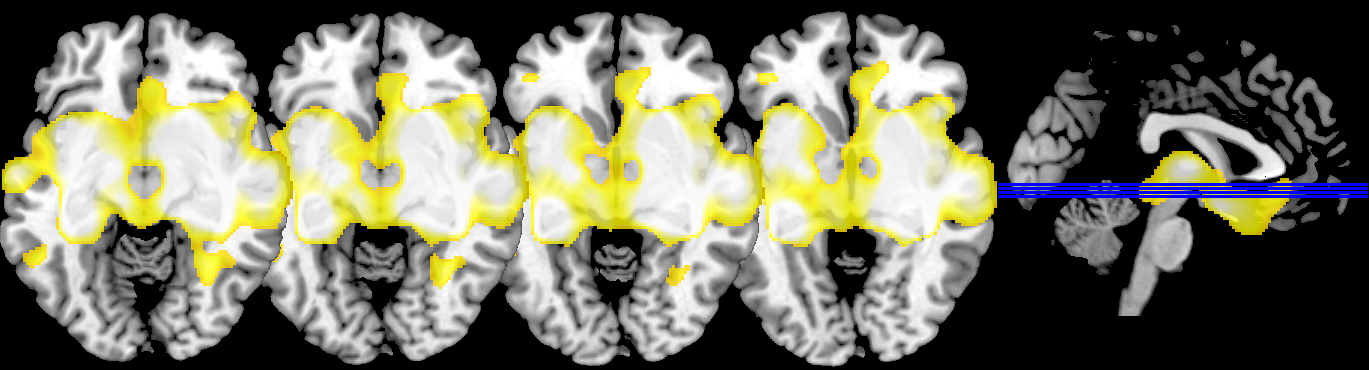
**

**
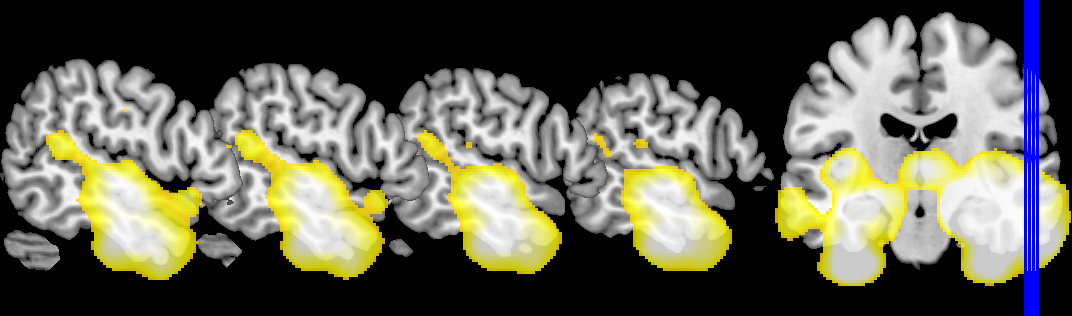
**

Supplementary Figure 3. Grey matter volume loss in our FTLD group with available research MRI scans overall (n=15) compared with elderly healthy control participants (n=15) with age as a covariate of no interest (SPM8, <http://www.fil.ion.ucl.ac.uk/spm/>, random effects model, 2-sample t-test). The upper panel displays axial slices in the subcallosal area and the lower panel sagittal slices through the right anterior temporal lobe using MRIcron (<http://people.cas.sc.edu/rorden/mricron/install.html> ([Rorden and Brett, 2000](#_ENREF_7))). Display threshold: uncorrected p = .001, 0 voxels. Regions surviving voxel-based familywise error correction at p=.05 were reported in Supplementary Table 4.

**Supplementary Table 1**

| Target consequence  Rating | Short-term consequences  *mean ± standard deviation* | Long-term consequences  *mean ± standard deviation* | p-value  (2-sided) |
| --- | --- | --- | --- |
| Pleasantness (-4 to +4) | -0.06±2.88 | -0.07±3.39 | .986 |
| Familiarity (1-7) | 4.23±1.03 | 3.10±1.21 | <.0001* |
| Likelihood (0-100) | 67.09±10.94 | 38.51±18.95 | <.0001* |

Normative data for means across 30 items in each condition of the consequences of social action task are reported. Significance at p=.05 2-sided using 2-sample t-tests are marked with *.

**Supplementary Table 2**

|  | | | Long-term consequences | Short-term consequences | Social concepts | Cambridge Naming | Cambridge Word to Picture Matching |
| --- | --- | --- | --- | --- | --- | --- | --- |
|  | Long-term consequences | rho | 1.0 | .52^*^ | .21 | .12 | .10 |
|  |  | p |  | .02 | .39 | .66 | .69 |
|  |  | n | 19 | 19 | 19 | 17 | 18 |
|  | Short-term consequences | rho | .52^*^ | 1.0 | .38 | .52^*^ | .67^**^ |
|  |  | p | .02 |  | .11 | .03 | .002 |
|  |  | n | 19 | 19 | 19 | 17 | 18 |
|  | Social concepts | rho | .21 | .38 | 1.0 | .60^*^ | .74^**^ |
|  |  | p | .39 | .11 |  | .01 | <.0001 |
|  |  | n | 19 | 19 | 19 | 17 | 18 |
|  | Cambridge Naming | rho | .12 | .52^*^ | .60^*^ | 1.0 | .95^**^ |
|  |  | p | .66 | .03 | .01 |  | <.0001 |
|  |  | n | 17 | 17 | 17 | 17 | 17 |
|  | Cambridge Word to Picture Matching | rho | 0.10 | .67^**^ | .74^**^ | .95^**^ | 1.0 |
|  |  | p | .69 | .002 | <.0001 | <.0001 |  |
|  |  | n | 18 | 18 | 18 | 17 | 18 |
| Spearman rho correlations between standard general semantic ([Bozeat et al., 2000](#_ENREF_2)) and experimental task measures in FTLD patients. All p-values are two-tailed. Spearman rho correlations significant at the p=.05 level are marked with *. ** corresponds to significance at the p=.01 level. | | | | | | | |
|  | | | | | | | |

**Supplementary Table 3**

|  | | | Long-term consequences | Short-term consequences | Social concepts | ACE-R  Total | ACE-R  Verbal | ACE-R  Visuo-spatial |
| --- | --- | --- | --- | --- | --- | --- | --- | --- |
|  | Long-term consequences | rho | 1.00 | .52* | .21 | .08 | .11 | -.03 |
|  |  | p |  | .02 | .39 | .74 | .64 | .89 |
|  | Short-term consequences | rho | .52* | 1.00 | .38 | .37 | .41 | -.14 |
|  |  | p | .02 |  | .11 | .12 | .08 | .58 |
|  | Social concepts | rho | .21 | .38 | 1.00 | .72** | .71** | .15 |
|  |  | p | .39 | .11 |  | .0006 | .0007 | .54 |
|  | ACE-R  Total | rho | .08 | .37 | .72** | 1.00 | .99** | .29 |
|  |  | p | .74 | .12 | .0006 |  | <.0001 | .22 |
|  | ACE-R  Verbal | rho | .11 | .41 | .71** | .99** | 1.00 | .22 |
|  |  | p | .64 | .08 | .0007 | <.0001 |  | .37 |
|  | ACE-R  Visuo-spatial | rho | -.03 | -.14 | .15 | .29 | .22 | 1.00 |
|  |  | p | .89 | .58 | .54 | .22 | .37 |  |

Spearman rho correlations between Total Addenbrooke’s Cognitive Examination(ACE)-R scores ([Mioshi et al., 2006](#_ENREF_5)), the sum of its verbal subtests (i.e. all subtests requiring the understanding and working with verbal materials), its visuo-spatial subtest, and experimental task measures in FTLD patients. All p-values are two-tailed. Spearman rho correlations significant at the p=.05 level are marked with *. ** corresponds to significance at the p=.01 level.

**Supplementary Table 4**

| Patient ID | Clinical Diagnosis | Classification |
| --- | --- | --- |
| FTD_10 | bvFTD w. SD features | Global Mixed |
| FTD_24 | bvFTD w. SD features | Global Mixed |
| FTD_03 | bvFTD w. features of SD+ | Primary ATL |
| FTD_12 | SD | Primary ATL |
| FTD_13 | SD | Primary ATL |
| FTD_14 | SD | Primary ATL |
| FTD_18 | SD w. bvFTD features | Primary ATL |
| FTD_20 | SD w. bvFTD features | Primary ATL |
| FTD_21 | SD | Primary ATL |
| FTD_22 | SD | Primary ATL |
| SD_10 | SD | Primary ATL |
| SD_11 | SD | Primary ATL |
| SD_12 | SD | Primary ATL |
| SD_13 | SD | Primary ATL |
| FTD_02 | bvFTD | Primary non-ATL |
| FTD_07 | Mixed PPA w. bvFTD features | Primary non-ATL |
| FTD_09 | bvFTD | Primary non-ATL |
| FTD_16 | bvFTD | Primary non-ATL |
| FTD_19 | bvFTD | Primary non-ATL |

Clinical diagnoses were lead symptom-based (i.e. based on the first and most prominent symptom according to caregiver history ([Zahn and Burns, 2017](#_ENREF_9))), this proved to best differentiate FTLD from Alzheimer’s disease in a neuropathological study ([Barber et al., 1995](#_ENREF_1)) and is in accordance with Lund Manchester diagnostic criteria ([Neary et al., 1998](#_ENREF_6)). Based on their clinical lead symptom as reported by carers (primary behavioural: non-ATL) vs. primary comprehension/naming: ATL), background neuropsychological tests (central semantic impairment yes: ATL, no: no ATL) and neuroimaging features (ATL atrophy on MRI: yes or no, global atrophy: yes or no), we classified patients into those with primary neurodegeneration of the ATLs (primary ATL group, n=12), those with primary neurodegeneration outside the ATL (primary non-ATL group, n=5, presumably involving frontal cortices, although we know that MRI is not sensitive to early changes ([Hodges, 2001](#_ENREF_4))) and those with global impairment or atrophy (n=2). SD = semantic dementia, bvFTD = behavioural variant of frontotemporal dementia, PPA = primary progressive aphasia, ATL = anterior temporal lobe. As expected, non-ATL patients had lower frontopolar relative to ATL grey matter volume (n=5, -.221±.088) compared to the ATL group (n=8, -.099±.042, t=3.4, p=.006, Mann-Whitney-U test: p=.045) and non-ATL patients also showed accordingly poorer performance on long-term consequences vs. social concept task conditions (n=5, -5.60±14.65) compared with the ATL group (n=12, 15.26±13.99, t=2.8, p=.014, Mann-Whitney-U test: p=.037). ^+^FTD_03 would have been classified as SD on the basis of her neuropsychological profile and MRI showing right ATL atrophy, but according to caregiver history presented primarily with inappropriate social behaviour over several years before developing impairments in naming, comprehension and object recognition.

**Supplementary Table 5**

| Hemi-sphere | Area | x | y | z | Brodmann Area | t -score |
| --- | --- | --- | --- | --- | --- | --- |
| R | Anterior parahippocampal gyrus | 23 | 3 | -14 | 34 | 10.99 |
| R | Midbrain | 18 | -23 | -9 | - | 8.45 |
| L | Hippocampus | -38 | -33 | -3 | - | 7.67 |
| L | Amygdala | -23 | 0 | -18 | - | 6.99 |
| R | Caudate tail | 35 | -33 | 0 | - | 6.96 |
| L | Anterior inferior temporal/fusiform gyrus | -36 | -7 | -39 | 20 | 6.57 |
| R | Anterior inferior temporal/fusiform gyrus | 39 | -8 | -36 | 20 | 6.30 |
| L | Claustrum | -32 | -1 | 7 | - | 6.19 |

Voxel-based morphometry analysis using a 2-sample t-test in SPM8 <http://www.fil.ion.ucl.ac.uk/spm/> to identify grey matter volume loss in the FTLD (n=15) vs. healthy elderly control group (n=15) whilst using age as a covariate of no interest. Only areas exceeding a voxel level threshold of P=0.001 uncorrected, 0 voxels and voxel-based familywise error-corrected P=.05 over the whole brain are reported (these were side maxima in one large cluster which was the only cluster surviving a familywise error corrected threshold of p =.05 over the whole brain). Montreal Neurological Institute coordinates were reported and transformed to Talairach coordinates (using Matthew Brett’s formula, http://www.mrc-cbu.cam.ac.uk/Imaging/ Common/mnispace.shtml) to derive anatomical labels using the Talairach atlas ([Talairach and Tournoux, 1988](#_ENREF_8)).

**Supplementary References**

Barber, R., Snowden, J.S., Craufurd, D. Frontotemporal Dementia and Alzheimers-Disease - Retrospective Differentiation Using Information from Informants. *J Neurol Neurosur Ps*, 1995. 59 (1): 61-70.

Bozeat, S., Lambon Ralph, M.A., Patterson, K., Garrard, P., Hodges, J.R. Non-verbal semantic impairment in semantic dementia. *Neuropsychologia*, 2000. 38 (9): 1207-1215.

Green, S., Lambon Ralph, M.A., Moll, J., Zakrzewski, J., Deakin, J.F., Grafman, J., Zahn, R. The neural basis of conceptual-emotional integration and its role in major depressive disorder. *Soc Neurosci*, 2013. 8 (5): 417-433.

Hodges, J.R. Frontotemporal dementia (Pick's disease): clinical features and assessment. *Neurology*, 2001. 56 (11 Suppl 4): S6-10.

Mioshi, E., Dawson, K., Mitchell, J., Arnold, R., Hodges, J.R. The Addenbrooke's Cognitive Examination Revised (ACE-R): a brief cognitive test battery for dementia screening. *International Journal of Geriatric Psychiatry*, 2006. 21 (11): 1078-1085.

Neary, D., Snowden, J.S., Gustafson, L., Passant, U., Stuss, D., Black, S., Freedman, M., Kertesz, A., Robert, P.H., Albert, M., Boone, K., Miller, B.L., Cummings, J., Benson, D.F. Frontotemporal lobar degeneration: a consensus on clinical diagnostic criteria. *Neurology*, 1998. 51 (6): 1546-1554.

Rorden, C., Brett, M. Stereotaxic display of brain lesions. *Behavioural Neurology*, 2000. 12: 191-200.

Talairach, J., Tournoux, P. *Co-Planar Stereotaxic Atlas of the Human Brain*. Thieme Medical Publishers: New York, 1988.

Zahn, R., Burns, A. Dementia disorders: an overview. in: Waldemar, G., Burns, A. (Eds.), *Alzheimer's Disease*. Oxford University Press: Oxford, 2017.

Zahn, R., Moll, J., Iyengar, V., Huey, E.D., Tierney, M., Krueger, F., Grafman, J. Social conceptual impairments in frontotemporal lobar degeneration with right anterior temporal hypometabolism. *Brain*, 2009. 132 (Pt 3): 604-616.
